# In this month’s Bulletin

**DOI:** 10.2471/BLT.17.000917

**Published:** 2017-09-01

In the editorial section, Melanie M Taylor et al. (610) describe how syphilis screening and treatment can be integrated with HIV services. Mary Malebranche et al. (611) propose measures to alleviate the difficulties faced by pregnant refugees. 

This month’s focus of the news section is the region of Africa. Tatum Anderson (614–615) reports on progress made in the provision of family planning services and Charles Shey Wiysonge explains to Fiona Fleck (616–617) how the Cochrane Collaboration contributes to evidence-based health care. 

## China

### Incentives for prevention

Wanyue Zhang et al. (658–662) report the effect of payments to providers for syphilis screening.

## Ethiopia 

### Treating tropical lymphoedema 

Kebede Deribe et al. (653–657) detail integrated care for lymphatic filariasis and podoconiosis. 

## South Africa

### After introducing pneumococcal vaccination

Alane Izu et al. (618–629) track hospitalizations of children for pneumonia. 

## Sri Lanka

### Human and canine rabies

A Pubudu De Silva et al. (647–652) describe a reporting system to improve recall and follow-up.

## Global

### Counting the benefits of vaccines

Sachiko Ozawa et al. (630–639) estimate the economic impact of vaccinations programmes in 73 countries.

### Choosing between 40,000 products

Cara S Kosack et al. (640–646) propose criteria for selecting diagnostic tests.

### Ethical implications of new technology

Tsung-Ling Lee et al. (663–664) argue for regulation of the stem cell industry.

